# DFL-MHC: MHC identification model based on dual-stage training and multi-view feature fusion

**DOI:** 10.3389/fgene.2026.1774569

**Published:** 2026-01-21

**Authors:** Yanjuan Li, Yiben Lin, Dong Chen

**Affiliations:** 1 College of Electrical and Information Engineering, Quzhou University, Quzhou, China; 2 School of Computer Science, Hangzhou Dianzi University, Hangzhou, China

**Keywords:** dimensionality reduction, dual stage training, feature extraction, major histocompatibility complex (MHC), protein identification

## Abstract

The major histocompatibility complex (MHC) is the central genetic basis of adaptive immune responses, it plays a crucial role in antigen presentation, immune surveillance, and susceptibility to various diseases. Therefore, accurate MHC identification is essential for both immunological research and clinical applications. Most existing methods still depend on manually engineered features or a single protein language model (PLM for short), these methods cannot perfectly capture complementary information across sequence lengths or across different PLMs. Furthermore, most existing methods often adopt conventional machine learning algorithms or simple multilayer perceptron (MLP) classifiers to construct identification model, they have no ability to model deep semantic dependencies within sequences. To overcome these limitations, we introduce an MHC identification model based on dual-stage training and multi-view feature fusion, termed DFL-MHC, a novel framework that unifies multi-sequence and multi-model views within a dual-stage training strategy. In the feature extraction stage, we design a cross-sequence and cross-model multi-view scheme. In this scheme, a protein sequence is truncated into two different residue sequences with a length of 1,022, two PLMs are respectively employed to extract features from the two different residue sequences, these extracted features are fused to represent the protein sequence. The dimensionality reduction algorithm is applied to the fused features and obtain the optimal feature subset. The optimal feature subset can fully capture complementary information across sequence lengths and across different PLMs. In the feature modeling stage, we construct a bi-directional long short-term memory (BiLSTM) network incorporating an attention mechanism to capture long-range dependencies and deep semantic dependencies within sequences. On the MHC identification task, DFL-MHC achieves better performance than the existing methods. It is demonstrated that the effectiveness of leveraging both multi-view feature fusion and dual-stage training to achieve accurate and reliable MHC identification.

## 1 Introduction

The major histocompatibility complex (MHC) refers to a cluster of genes situated on the short arm of human chromosome 6. MHC-encoded products are pivotal to the processes of antigen presentation and adaptive immune responses (Kubiniok et al., 2022; Wassenaar et al., 2024). Due to the high polymorphism of MHC genes, different alleles exhibit substantial variations in immune responses and disease susceptibility, which not only constitute the molecular basis of organ transplant rejection but are also closely associated with autoimmune diseases and tumor immunity (Tsai and Santamaria, 2013). Therefore, rapid and accurate MHC identification is of great importance for both fundamental research and clinical applications (Neefjes et al., 2011; Trowsdale and Knight, 2013).

Early identification of MHC molecules primarily relied on serological assays and cytotoxicity tests. Although these experimental approaches provided relatively high accuracy, they were time-consuming, costly, and constrained by laboratory conditions. Then, these experimental approaches are difficult to handle large-scale biological data (Middleton, 2005; Choi et al., 2024). With the advancement of computational biology and machine learning (Mohapatra et al., 2025; Wang et al., 2024), researchers began to explore computational approaches to identify MHC for improving efficiency. For instance, Li et al. (Li et al., 2019) proposed ELM-MHC, which encoded protein sequences with manually engineered feature strategies, such as SVMProt 188D (Ali et al., 2025), bag of ngram (BonG) (Wisky et al., 2024), and information theory (IT). The mixed features were trained by extreme learning machine (ELM), then the MHC identification model was constructed, and the model has better performance. Chen et al. (Chen and Li, 2022) further improved ELM-MHC and introduced a novel model named PredMHC, which integrated multiple manually extracted protein features including 188D, APAAC, KSCTriad, CKSAAGP, and PAAC to represent protein sequences. The fused features are applied to train three classifiers including SMO, SGD and random forests, then the voting of the tree models is used as the identification result. Although these methods gained better performance in MHC identification, they all adopted manually engineered features. Then, these methods cannot fully capture deep semantic dependencies within sequences.

In the past few years, the emergence of deep learning has significantly advanced MHC identification. Large-scale language models have achieved groundbreaking progress in natural language processing through self-supervised learning on massive datasets of unlabelled textual data, thereby facilitating the automatic acquisition of underlying syntactic and semantic rules (Zou K. et al., 2023; Devlin et al., 2019; Ren et al., 2025). Building on this paradigm, protein language models (PLMs) extend the idea to biological sequences (Luo et al., 2025; Soylu and Sefer, 2024). By pretraining on massive protein sequence databases, PLMs eliminate the need for labor-intensive handcrafted feature design and can generate high-dimensional, biologically meaningful representations that capture the underlying grammar and semantics of proteins (Brandes et al., 2022; Brandes et al., 2023; Wu et al., 2025; Li et al., 2021). As a representative model, Evolutionary Scale Modeling (ESM) (Rives et al., 2021; Xu and Science, 2024) employ Transformer-based architectures to capture contextual dependencies among amino acid residues during unsupervised pretraining, it has gained outstanding performance in many tasks including protein classification, functional annotation, and structure prediction (Mu et al., 2025; Cheng et al., 2025; Yuan et al., 2024; Ahmed et al., 2025), and it has also facilitated the modeling of complex immune-related problems (Hashemi et al., 2023; Yadav et al., 2024). Based on PLM, Cai et al. (2024) proposed a MHC identification method called ESM-MHC. ESM-MHC extracts features using ESM-1b, and then carries out PCA for the purpose of dimensionality reduction, finally employs multilayer perceptron (MLP) classifier to construct identification model. Although ESM-MHC obtained better performance, it only inputted large model embedding vectors into MLP for prediction. That is, ESM-MHC only used a single large language model and simple MLP classification, it failed to fully utilize the potential of deep models in sequence dependent modeling and feature interaction. To solve limited expressiveness of a single large language model, many researchers attempt to fuse features of multiple large language models to improve accuracy (Watanabe et al., 2024; Barabucci et al., 2024).

Meanwhile, the development of deep learning has introduced a variety of alternatives to MLPs for sequence modeling. Long short-term memory (LSTM) and gated recurrent unit (GRU), two typical architectures of recurrent neural networks (RNNs), can effectively capture sequential dependencies (Pawar, 2025; Chung et al., 2014; Zulfiqar et al., 2024; Qiao et al., 2024; Xie H. et al., 2025; Yan et al., 2024), while attention mechanisms adaptively allocate weights to highlight key features, offering unique advantages in modeling long-range dependencies (Vaswani et al., 2017). Several studies have combined LSTM with attention mechanisms to simultaneously preserve local temporal information and global dependencies during sequence modeling, thus yielding more comprehensive and fine-grained representations of protein sequences (Fan and Xu, 2024; Nallapareddy and Dwivedula, 2021; Wang et al., 2023).

Motivated by these advances, we propose DFL-MHC, a model that integrates multi-view feature fusion with a dual-stage training strategy. In the first stage, a protein sequence is truncated into two different amino acid sequences with a length of 1,022 from the first direction and the last direction, respectively. ESM-1b and ESM-2 are respectively employed to extract features from two different amino acid sequences. Thus, for a protein sequence, four features are obtained. Their combinational features across sequence and across model is reduce to the optimal feature subset based on PCA and MLP. In the second stage, we input the optimal features into a deep framework that incorporates an attention mechanism into a bidirectional LSTM (BiLSTM) model. The deep framework can capture long-range dependencies and dynamically highlight critical information. Through this design, DFL-MHC is intended to provide a more effective framework for advancing MHC identification.

## 2 Materials and methods

### 2.1 Framework of DFL-MHC

**FIGURE 1:**
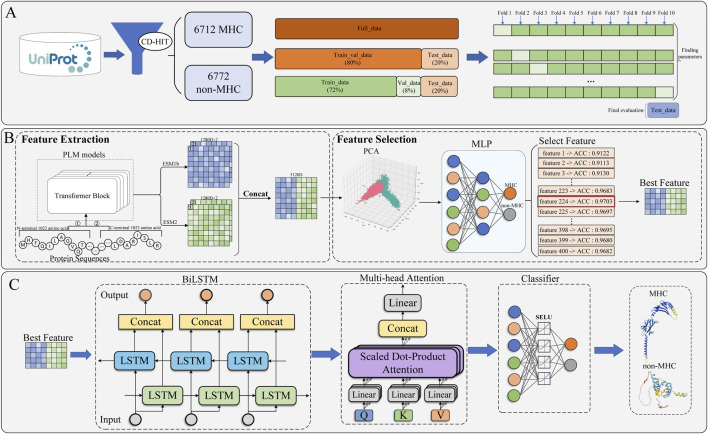
The framework of DFL-MHC. **(A)** Data collection procedure for MHC and non-MHC samples. **(B)** Initial feature screening, consisting of feature extraction and feature selection. **(C)** Model training process.

In this study, we present DFL-MHC, a model that employs dual-stage feature learning to achieve multi-view fusion. Figure 1 illustrates the overall workflow.

As shown in Figure 1A, during the data acquisition stage, MHC and non-MHC protein sequences were obtained from the UniProt database (Kulyyassov, 2022) and sequence redundancy was reduced through the application of the CD-HIT tool. Finally, we partitioned the full dataset into Train_val_data, Test_data in the ratio of 8:2, the Train_val_data is further split into Train_data (72%) and Val_data (8%) within each fold.

The first stage is feature extraction and selection, as illustrated in Figure 1B. We employed two PLMs, ESM-1b and ESM-2, to generate embeddings from a multi-model perspective. Considering the input length limitations of ESM models, protein sequences were segmented into multiple intervals, and features were extracted from each segment to obtain cross-sequence representations. By further combining these representations across models, we constructed comprehensive embeddings through feature concatenation. However, directly training on such high-dimensional features would not only incur excessive computational cost but also increase the risk of overfitting. In response, we employed PCA to reduce the dimensionality of the embeddings, preserving the optimal features as the foundation for the subsequent deep modeling stage.

The second stage is model training, as illustrated in Figure 1C. We designed a deep modeling architecture that integrates a BiLSTM network with a multi-head attention mechanism, which can effectively capture both contextual dependencies and global representations. Finally, the classification module consisted of two linear layers with a scaled exponential linear unit (SELU) activation function inserted between them, which jointly produced the prediction results for MHC classification.

### 2.2 Dataset

In our experiments, we used the dataset provided by Li et al., (2019). Their data came from UniProt and had been run through CD-HIT to reduce redundancy among similar sequences. 6,712 of these were MHC samples, while 6,772 were non-MHC. In total, the dataset consists of 13,488 protein sequences. It was divided into Train_val_data and Test_data with an 8:2 ratio as seen in Figure 1. There were 10,790 sequences in the training dataset, and 2,698 sequences were left for testing.

### 2.3 Feature extraction

As a core and indispensable step, feature extraction plays a decisive role in building effective classification models. Traditional approaches mainly rely on manually engineered descriptors, such as physicochemical properties, structural information, or statistical indices, and then integrate multiple descriptors to form a composite feature set (Zou X. et al., 2023; Zhu et al., 2023; Chen et al., 2025). However, these methods face inherent limitations. On the one hand, manual feature engineering depends heavily on prior knowledge and is insufficient to capture the latent higher-order information embedded in protein sequences. On the other hand, redundancy or noise among heterogeneous descriptors may compromise the model’s generalization ability. In contrast, PLMs, pre-trained on large-scale protein sequence databases, are capable of automatically learning contextual dependencies and latent semantic representations of amino acid residues. This avoids the need for laborious handcrafted feature design and demonstrates superior performance in capturing both global and local sequence patterns. In this work, we adopt the ESM-1b and ESM-2 for feature extraction and further introduce cross-model and cross-sequence perspectives to leverage the complementary strengths of different models and sequence segments while preserving information integrity. A brief introduction of the selected ESM models and our fusion strategy is provided below.

#### 2.3.1 ESM-1b

To capture the global dependencies of MHC sequences, we used ESM-1b to encode protein sequence. ESM-1b is trained on the high-diversity UR50/S dataset from UniRef50, it has 650 million parameters (Rives et al., 2021). Unlike the conventional sequence model, ESM-1b stacks 33 Transformer layers and can generates embedding representations and attention weights for protein sequences. One of the key features of ESM-1b is that it learns the interchangeability of amino acids through Mask Language Modeling (MLM) tasks, ESM-1b is less sensitive to noise.

#### 2.3.2 ESM-2

ESM-2 is also used in this paper. The model needs to predict the type of masked residue based on context, rather than directly memorizing the complete sequence. Architecturally, optimizations in attention mechanisms and layer normalization contribute to enhanced representational power and training stability. ESM-2 has achieved superior accuracy and robustness in tasks such as protein structure prediction (Cheng et al., 2025), residue contact inference, and functional site identification, particularly excelling at capturing local structural motifs and short-range dependencies.

#### 2.3.3 Integration of ESM-1b and ESM-2

ESM-1b and ESM-2 are two protein language models based on transformer. However, they can generate complementary feature representations based on their different architectural inductive biases and training strategies. In detail, ESM-1b was trained on the early UniRef50 database, while ESM-2 was trained on an extended UniRef dataset. The convergence points in the loss landscape of ESM-1b distinguishes from one of ESM2, therefore, they capture different subsets of evolutionary semantics.

In addition, ESM1b and ESM2 employs different positional encoding mechanisms. ESM1b can capture the absolute positional information of amino acid residues by using learned positional embeddings. Therefore, ESM1b is sensitive to fixed-position sequence motifs. Contrast to ESM1b, ESM2 mathematically represent the relative distances between residues by employing Rotary Positional Embeddings (RoPE). ESM2 can effectively model the translation-invariant geometric relationships and long-range dependencies and characterize the flexible peptide-binding groove. Based on these differences, the integration of ESM-1b and ESM-2 does not merely increase dimensionality but integrates two complementary views of protein biology: the absolute coordinate–based motif recognition and the relative geometry-based structural inference.

### 2.4 Feature selection

In general, the combination of multiple feature can better represent protein sequences. In practice, when the quantity of features far surpasses that of samples, the model trained on these features will be overfitting. Moreover, high dimensional inputs can also increase computational load and slow down training speed. PCA is a classic dimensionality reduction method, which achieves the projection of high-dimensional data onto a series of orthogonal components by means of linear transformation (Souza, 2025). In this paper, PCA is used to reduce the dimension of features, then use MLP to select the optimal feature subset, as shown in Figure 1B. Section 3.2 shows the experimental results.

### 2.5 Model training

As shown in Figure 1C, we employed a hybrid architecture to train MHC identification model. By combining BiLSTM and multi-head attention mechanisms, the proposed model is able to identify and extract local and global dependencies inherent in protein sequences. In detail, BiLSTM is an extension of standard LSTM, it processes information from forward and backward directions. Forward LSTM models parse input sequences following their inherent chronological sequence, whereas backward LSTM networks process the same sequences in the reversed order, ultimately concatenating or adding their outputs to form a more comprehensive contextual representation. The multi-head attention mechanism can independently run multiple attention heads, then used different linear transformation matrix to parallelly compute attention distributions of different subspaces. It can enhance the perception of global dependencies and facilitates richer feature interactions. In the final classification stage, we designed a classifier consisting of a linear layer and a SELU activation. By providing self-normalization, SELU mitigates vanishing and exploding gradients, improving training stability and convergence speed. The classifier ultimately outputs MHC type predictions through a binary classification layer.

### 2.6 Evaluation metrics

In this paper, we used four commonly adopted evaluation metrics for assessing model performance, encompassing Accuracy (ACC), Specificity (SP), Sensitivity (SN), and Matthews Correlation Coefficient (MCC) (Zeng et al., 2025; Xie X. et al., 2025). Accuracy measures the proportion of correctly predicted examples among all examples and serves as the most intuitive indicator of overall classification performance. A higher ACC generally indicates stronger overall discriminative ability; however, it may be biased in cases of imbalanced class distribution. It is defined as follows (Huang et al., 2025; Zhu et al., 2024; Huang et al., 2024; Liu et al., 2019).

$$ACC=\frac{TP+TN}{TP+FP+TN+FN}$$

where TP, TN, FP, and FN represent the numbers of true positives, true negatives, false positives, and false negatives, respectively.

Specificity evaluates the model’s ability to correctly identify negative samples, i.e., the proportion of actual negative samples correctly classified as negative. A higher SP indicates effective reduction of false-positive predictions.

$$SP=\frac{TN}{TN+FP}$$

Sensitivity measures the model’s capability to correctly identify positive samples, i.e., the proportion of actual positive samples correctly classified as positive. A higher SN reflects effective capture of target class samples.

$$SN=\frac{TP}{TP+FN}$$

MCC considers all four values (TP, TN, FP, FN) and provides a robust and reliable performance measure even under imbalanced class distributions. Its range is [-1, 1], where 1 indicates perfect classification, 0 corresponds to random prediction, and −1 denotes complete misclassification. MCC is calculated as.

$$MCC=\frac{TP\times TN-FP\times FN}{\sqrt{\left(TP+FP\right)\left(TP+FN\right)\left(TN+FP\right)\left(TN+FN\right)}}$$

## 3 Result and discussion

### 3.1 Comparative analysis of different features

Feature extraction is a critical step in identifying MHC. Traditional feature extraction methods mainly rely on manually designed feature descriptors, such as AAC (Barnum et al., 2024), CTriad, DDE and CKSAAP (Usman and Lee, 2019). They suffer from the reliance on prior knowledge, unable to capture long-range protein sequence interactions and have high-dimensional noise.

We compared three encoding schemes: traditional handcrafted descriptors including (QSOrder) (Chou, 2000), CKSAAP, AAC etc., single PLM embeddings, and our fusion features. We validated these methods using 10-fold cross-validation and independent testing, with details provided in Tables 1, 2.

**TABLE 1:** Performance of different features on training set.

Feature	ACC	SP	SN	MCC
AAC	0.9171	0.9187	0.9168	0.8355
DDE	0.9163	0.9178	0.9160	0.8338
Ctriad	0.9170	0.9189	0.9166	0.8355
CTDC	0.9182	0.9198	0.9178	0.8376
CTDT	0.9154	0.9168	0.9161	0.8319
QSOrder	0.9155	0.9169	0.9152	0.8321
CKSAAP	0.9158	0.9175	0.9155	0.8330
AAC + CKSAAP	0.9170	0.9184	0.9166	0.8351
Ctriad + DDE	0.9162	0.9179	0.9159	0.8337
CKSAAP + DDE	0.9168	0.9185	0.9164	0.8350
CTDC + CTDT + CTDD	0.9185	0.9201	0.9182	0.8383
ACC + QSOrder + DDE	0.9159	0.9178	0.9156	0.8333
ESM-1b	0.9503	0.9506	0.9508	0.9014
ESM-1b × 2	0.9629	0.9632	0.9634	0.9266
ESM-2	0.9496	0.9500	0.9501	0.9001
ESM-2 × 2	0.9537	0.9539	0.9541	0.9080
ESM-1b + ESM-2	0.9644	0.9645	0.9647	0.9293
ESM-1b + ESM-2 × 2	0.9648	0.9652	0.9646	0.9298
ESM-1b + ESM-2 × 2	0.9673	0.9676	0.9672	0.9348
ESM-1b × 2 + ESM-2 × 3	0.9685	0.9683	0.9679	0.9362
ESM-1b × 3 + ESM-2 × 2	0.9665	0.9668	0.9663	0.9331
ESM-1b × 2 + ESM-2 × 2	0.9703	0.9707	0.9702	0.9409

**TABLE 2:** Performance of different features on test set.

Feature	ACC	SP	SN	MCC
AAC	0.9081	0.9091	0.9087	0.8178
DDE	0.9055	0.9067	0.9062	0.8129
Ctriad	0.9081	0.9092	0.9088	0.8180
CTDC	0.9081	0.9092	0.9087	0.8179
CTDT	0.9125	0.9142	0.9133	0.8275
QSOrder	0.9114	0.9125	0.9121	0.8246
CKSAAP	0.9059	0.9074	0.9066	0.8148
AAC + CKSAAP	0.9051	0.9069	0.9062	0.8131
Ctriad + DDE	0.9055	0.9052	0.9047	0.8100
CKSAAP + DDE	0.9051	0.9062	0.9058	0.8120
CTDC + CTDT + CTDD	0.9118	0.9133	0.9126	0.8259
ACC + QSOrder + DDE	0.9129	0.9150	0.9138	0.8287
ESM-1b	0.9506	0.9513	0.9504	0.9017
ESM-1b × 2	0.9639	0.9643	0.9637	0.9280
ESM-2	0.9515	0.9522	0.9513	0.9035
ESM-2 × 2	0.9534	0.9539	0.9532	0.9071
ESM-1b + ESM-2	0.9626	0.9626	0.9629	0.9255
ESM-1b × 2 + ESM-2	0.9629	0.9630	0.9633	0.9263
ESM-1b + ESM-2 × 2	0.9644	0.9645	0.9647	0.9292
ESM-1b × 2 + ESM-2 × 3	0.9685	0.9686	0.9688	0.9374
ESM-1b × 3 + ESM-2 × 2	0.9678	0.9678	0.9681	0.9359
ESM-1b × 2 + ESM-2 × 2	0.9689	0.9690	0.9692	0.9382

To evaluate the effectiveness of sequence truncation and integration strategies, we defined three extraction paradigms:

1. Single-view (×1): Extracting features only from the first 1,022 residues (N-terminus) by ESM-1b or ESM2, denoted by ESM-1b or ESM2.
2. Dual-view (×2): Combination of the embedding respectively extracted from the N-terminus (first 1,022 residues) and C-terminus (last 1,022 residues) by ESM-1b or ESM2, denoted by ESM-1b×2 or ESM2 × 2.
3. Tri-view (×3): Concatenation of features extracted from N-terminus, C-terminus, and the central sequence region ESM-1b or ESM2, denoted by ESM-1b×3 or ESM2 × 3.

In 10-fold cross-validation, ESM1b achieved 0.9503 accuracy compared to 0.9171 of AAC, proving that PLM features are superior to manual ones. We also observed that single PLMs is inferior than combined ones. As integrating ESM2 with ESM1b yielded an accuracy of 0.9644 and an MCC of 0.9293, both outperforming individual models. The best one is ESM2 × 2 and ESM1b × 2, with an accuracy of 0.9703, specificity of 0.9707, sensitivity of 0.9702, and MCC of 0.9409. This proves that multi-view fusion is the main reason for these good results. It is also shown that extending from the dual-view to the tri-view strategy degrades performance, it is likely due to redundancy or noise introduced by the central region. Therefore, we used the ESM-2 × 2 + ESM-1b × 2.

**FIGURE 2:**
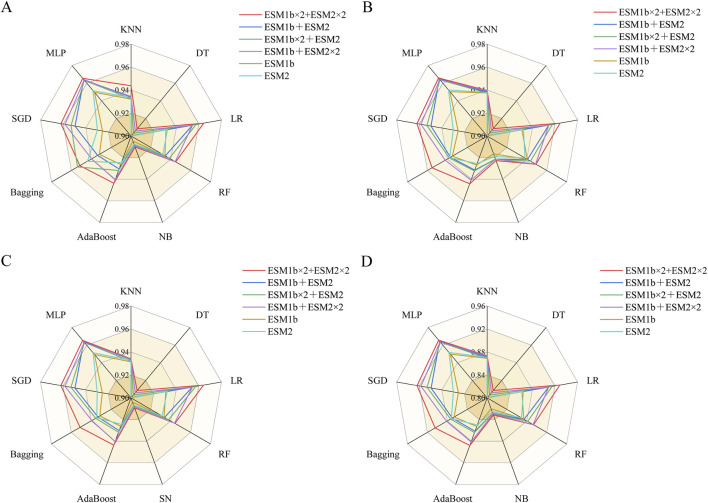
Comparison of features from different PLMs across multiple classifiers according to different indicators. **(A)** ACC indicator. **(B)** SP indicator. **(C)** SN indicator. **(D)** MCC indicator.

Figure 2 shows the performance of different ESM features across multiple classifiers. It indicates that single ESM features have limitations in fully capturing protein sequence information. And combining pre-trained ESM with multidimensional sequence representations shows richer embeddings, having ability to effectively compensate for the deficiencies of individual features. In general, multiple feature fusion can better capture sequence patterns and improve identification performance.

The fusion features of multiple pre-trained ESM feature extracted from different part of sequence can better encode MHC protein sequences, having ability to effectively compensate for the shortcoming of individual features. It is shown in the experiment that Multi feature fusion can better capture sequence patterns and improve identification performance.

### 3.2 Effectiveness of dimensionality reduction strategy

We conducted an experiment to validate our dimensionality reduction strategy. The multi-model fusion strategy resulted in a 5,120-dimensional feature space. Instead of using the entire feature set, the feature was reduced to 1 to 400 principal components, as mentioned below, and tested with MLP.

**FIGURE 3:**
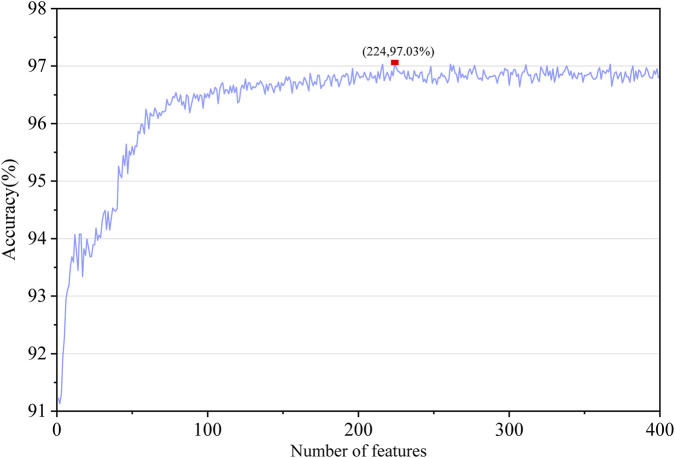
Model accuracies with features of different PCA dimensions.

Figure 3 shows the feature performance of different PCA dimensions. In particular, the accuracy curve shoots up steeply from dimension 1 to 180, which demonstrates that PCA is incorporating the helpful discriminative features. As shown in Figure 3, the 224-dimensional features achieved the highest accuracy, therefore we set the dimension of features to 224.

**FIGURE 4:**
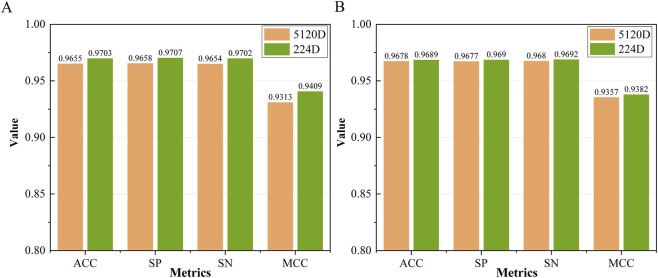
Performance comparison before and after dimensionality reduction. **(A)** Training dataset, **(B)** test dataset.

To further to verify the generalization ability of 224-dimensional features, we compared it with 5120-dimensional features (no PCA dimensional reduction) on the training dataset and test dataset shown in Figure 4. Across both training and test datasets, the performance of models using 224-dimensional features consistently exceeded that of models based on the original 5120-dimensional representations, confirming that the proposed dimensionality reduction strategy is both rational and effective for MHC prediction tasks.

### 3.3 Comparative analysis of different classifiers

During the feature selection stage, it is essential to choose an appropriate classifier to preliminarily evaluate the reduced features, ensuring that the rich multi-model and cross-segment information is effectively preserved. We compared MLP with eight widely used and high-performance classifiers, including Naive Bayes (NB) (Pajila et al., 2023), AdaBoost (Tien Bui et al., 2016), Random Forest (RF), Decision Tree (DT), Bagging, K-Nearest Neighbors (KNN), Logistic Regression (LR), and the Stochastic Gradient Descent Classifier (SGDClassifier) (Balaji et al., 2024).

The performance of these classifiers is summarized in Tables 3, 4. It is shown that the MLP consistently achieved the highest value across all metrics. Therefore, we used MLP as the classifier for feature dimension reduction.

**TABLE 3:** Performance of nine different classifiers on training set.

Feature	ACC	SP	SN	MCC
KNN	0.9347	0.9395	0.9341	0.8735
DT	0.9082	0.9083	0.9083	0.8166
LR	0.9642	0.9645	0.9641	0.9286
NB	0.9101	0.9227	0.9091	0.8317
RF	0.9449	0.9491	0.9443	0.8934
AdaBoost	0.9438	0.9443	0.9437	0.888
Bagging	0.9515	0.9533	0.9512	0.9045
SGD	0.9625	0.9626	0.9624	0.925
MLP	0.9655	0.9658	0.9654	0.9313

**TABLE 4:** Performance of nine different classifiers on test set.

Feature	ACC	SP	SN	MCC
KNN	0.9322	0.9358	0.9333	0.8691
DT	0.9092	0.9091	0.9093	0.8184
LR	0.9637	0.9641	0.9642	0.9282
NB	0.9025	0.9137	0.9045	0.8181
RF	0.9411	0.9444	0.9422	0.8865
AdaBoost	0.9396	0.9396	0.9398	0.8794
Bagging	0.947	0.9486	0.9478	0.8963
SGD	0.9663	0.9672	0.9669	0.9341
MLP	0.9678	0.9677	0.968	0.9357

**FIGURE 5:**
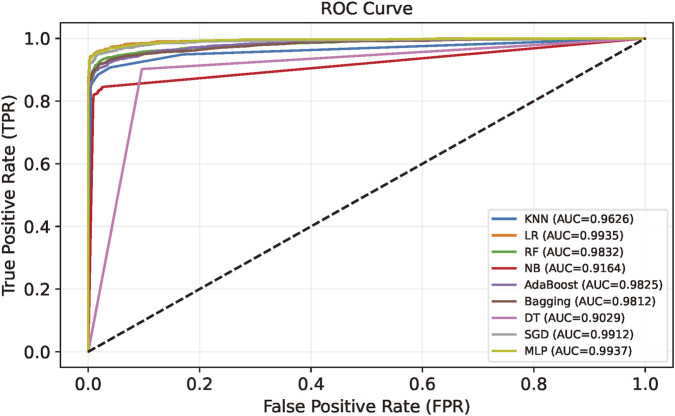
ROC curves of nine different classifiers on the test set.

Figure 5 shows the ROC curves on the test set. The MLP almost achieved the highest value throughout the entire curve, reaching an AUC of 0.9937. Its trajectory leans strongly toward the upper-left corner, indicating good stability on unseen samples. Taken together, these observations suggest that the MLP generalizes better than the other candidates and fits the requirements of the MHC identification task.

### 3.4 Ablation results of different modules

**FIGURE 6:**
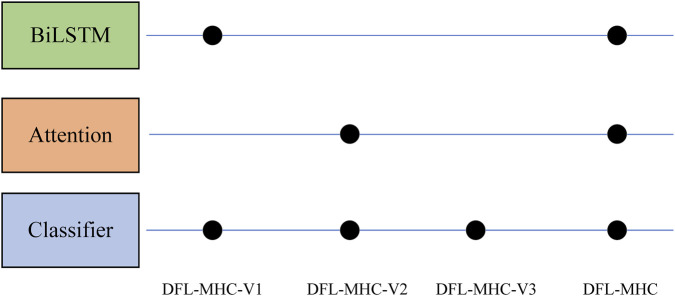
Variants of the DFL-MHC model.

To assess the impact of each module on the overall performance, we conducted a series of ablation experiments. In detail, we respectively removed BiLSTM and Attention components from the DFL-MHC architecture, resulting in three different variants (see Figure 6).

1. DFL-MHC-V1: This version does not have the BiLSTM module. In order to determine whether the model performs poorly when bidirectional dependency modeling is removed.
2. DFL-MHC-V2: In this case, the Attention mechanism is excluded. The goal is to determine whether identifying important residues is critical to the model’s final accuracy.
3. DFL-MHC-V3: BiLSTM and Attention were taken out, leaving only the final classifier to perform prediction tasks.

**FIGURE 7:**
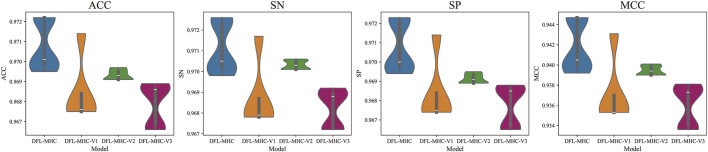
10-fold cross-validation results of DFL-MHC model variants on training dataset.

**FIGURE 8:**
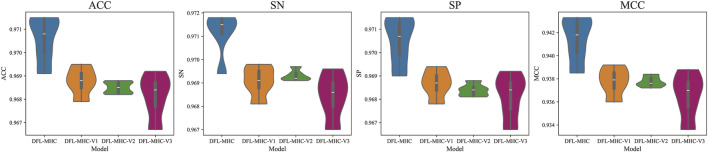
Test set evaluation of DFL-MHC model variants.

It is evident from Figures 7, 8 that the whole DFL-MHC model performs better than every variant in the indicator of ACC, SN, SP, and MCC. The Performance of DFL-MHC-V1 drastically decreased when the module of BiLSTM was removed, particularly in the indicator of SN. This demonstrates that BiLSTM is required to model sequential dependencies. Results for DFL-MHC-V2 indicate that SP and overall stability suffer when the Attention module is skipped. By combining BiLSTM and Attention module, the model may simultaneously identify important residue properties and long-range dependencies, improving accuracy.

Lastly, the performance of DFL-MHC-V3 shows the largest decline, in which both BiLSTM and Attention module are removed. It is also shown in Figures 7, 8 that all metrics significantly declined when both modules were removed, demonstrating that these two modules are mutually supportive. In general, experimental results show that BiLSTM and Attention module can improve the performance of DFL-MHC.

### 3.5 Comparative analysis with other methods

To Examine the performance of the proposed approach in MHC identification, we compared DFL-MHC against three representative models.

**FIGURE 9:**
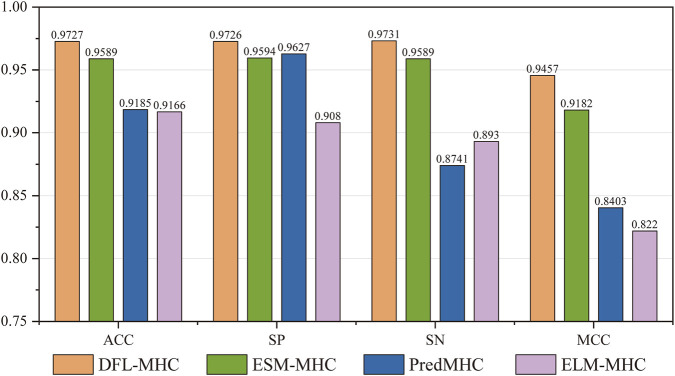
Comparison of DFL-MHC performance with existing methods.

Figure 9 shows the comparing results. DFL-MHC received scores of 0.9727 for ACC, 0.9726 for SP, 0.9731 for SN, and 0.9457 for MCC on the test set. The MCC of our DFL-MHC is 0.9457, obtaining a 2.9% improvement over the best-performing baseline, ESM-MHC. It is verified that our DFL-MHC can better differentiate positive examples from negative ones. In summary, DFL-MHC gained the best performance in identifying MHC among the existing methods.

## 4 Conclusion

In this paper, we introduced a MHC identification model named DFL-MHC. It consists of two stage, including feature extraction stage and feature modeling stage. During the feature extraction stage, we use the combinational features across sequences and across protein language models to encode protein sequences. In detail, for a given protein sequence, we respectively extract ESM-1b or ESM-2 embedding from the first 1,022 amino acids and the last 1,022 amino acids. Then four embeddings are combined to encode a protein sequences. The combinational embedding is reduced to an optimal feature subset based on PCA and MLP. In the feature modeling stage, DFL-MHC integrates BiLSTM networks with multi-head attention mechanism, it can simultaneously capture local sequence patterns and global dependencies. Furthermore, the SELU activation function enhances training stability and improves generalization performance.

Experimental results indicate that: (1) the combinational features across sequences and across protein language model have better performance than single protein language model or extracting features only on the first 1,022 amino acids. (2) the feature selectin module can improve identification performance. (3) ablation experiment of BiLSTM and multi-head attention verifies their contributions to performance enhancement and training stability. (4) the experimental results comparing with other methods verify that DFL-MHC consistently outperforms existing methods on all indicators, highlighting the effectiveness of the dual-stage training strategy and combinational feature across sequences and across protein language models for MHC identification.

## Data Availability

The original contributions presented in the study are included in the article/supplementary material, further inquiries can be directed to the corresponding author. Publicly available datasets were analyzed in this study. This data can be found here: https://github.com/benl1n/DFL-MHC.

## References

[B1] Ahmed F. S. Aly S. Liu X. (2025). EPI-HAN: identification of enhancer promoter interaction using hierarchical attention network. Curr. Bioinforma. 20 (5), 379–391. 10.2174/0115748936294743240524113731

[B2] Ali F. Ibrahim N. Alsini R. Masmoudi A. Alghamdi W. Alkhalifah T. (2025). “Comprehensive analysis of computational models for prediction of anticancer peptides using machine learning and deep learning,” in Archives of Computational Methods in Engineering (NY, United States: Springer), 1–21.

[B3] Balaji R. J. Manoj J. Kan V. (2024). “Brain tumor detection: deploying stochastic gradient descent classifier in a web app,” in 2024 Ninth International Conference on Science Technology Engineering and Mathematics (ICONSTEM), 1–10. 10.1109/iconstem60960.2024.10568624

[B4] Barabucci G. Shia V. Chu E. Harack B. Laskowski K. Fu N. (2024). Combining multiple large language models improves diagnostic accuracy. NEJM AI 1 (11). 10.1056/aics2400502

[B5] Barnum T. P. Crits-Christoph A. Molla M. Carini P. Lee H. Ostrov N. (2024). Predicting microbial growth conditions from amino acid composition. bioRxiv. 10.1101/2024.03.22.586313

[B29] Brandes N. Goldman G. Wang C. H. Ye C. J. Ntranos V. (2023). Genome-wide prediction of disease variant effects with a deep protein language model. Nat. Genet. 55 (9), 1512–1522. 10.1038/s41588-023-01465-0 37563329 PMC10484790

[B6] Brandes N. Ofer D. Peleg Y. Rappoport N. Linial M. (2022). ProteinBERT: a universal deep-learning model of protein sequence and function. Bioinformatics 38, 2102–2110. 10.1093/bioinformatics/btac020 35020807 PMC9386727

[B7] Cai J. Li Y. Chen D. (2024). “ESM-MHC: an improved predictor of MHC using ESM protein language model,” in Proceedings of the 2024 16th International Conference on Bioinformatics and Biomedical Technology, 88–95. 10.1145/3674658.3674674

[B8] Chen D. Li Y. (2022). PredMHC: an effective predictor of major histocompatibility complex using mixed features. Front. Genet. 13, 875112. 10.3389/fgene.2022.875112 35547252 PMC9081368

[B9] Chen Y. Wang Z. Wang J. Chu Y. Zhang Q. Li Z. A. (2025). Self-supervised learning in drug discovery. Sci. China Inf. Sci. 68 (7), 170103. 10.1007/s11432-024-4453-4

[B10] Cheng L. Lu W. Xia Y. Lu Y. Shen J. Hui Z. (2025). ProAttUnet: advancing protein secondary structure prediction with deep learning via U-Net dual-pathway feature fusion and ESM2 pretrained protein language model. Comput. Biol. Chem. 118. 10.1016/j.compbiolchem.2025.108429 40288255

[B11] Choi H. Choi E.-J. Kim H. J. Baek I. C. Won A. Park S. J. (2024). A walk through the development of human leukocyte antigen typing: from serologic techniques to next-generation sequencing. Clin. Transplant. Res. 38 (4), 294–308. 10.4285/ctr.24.0055 39658458 PMC11732764

[B12] Chou K. (2000). Prediction of protein subcellular locations by incorporating quasi-sequence-order effect. Biochem. Biophys. Res. Commun. 278 (2), 477–483. 10.1006/bbrc.2000.3815 11097861

[B13] Chung J. Gulcehre C. Cho K. Bengio Y. (2014). Empirical evaluation of gated recurrent neural networks on sequence modeling. Eprint Arxiv.

[B14] Devlin J. Chang M. W. Lee K. Toutanova K. (2019). BERT: pre-training of deep bidirectional transformers for language understanding. ArXiv.

[B15] Fan Z. Xu Y. (2024). Predicting the functional changes in protein mutations through the application of BiLSTM and the self-attention mechanism. Ann. Data Science (3), 11. 10.1007/s40745-024-00530-7

[B16] Hashemi N. Hao B. Ignatov M. Paschalidis I. C. Vakili P. Vajda S. (2023). Improved prediction of MHC-peptide binding using protein language models. Front. Bioinforma. 3, 1207380. 10.3389/fbinf.2023.1207380 37663788 PMC10469926

[B18] Huang Z. Guo X. Qin J. Gao L. Ju F. Zhao C. (2024). Accurate RNA velocity estimation based on multibatch network reveals complex lineage in batch scRNA-seq data. BMC Biol. 22 (1), 290. 10.1186/s12915-024-02085-8 39696422 PMC11657662

[B19] Huang Z. Xiao Z. Ao C. Guan L. Yu L. (2025). Computational approaches for predicting drug-disease associations: a comprehensive review. Front. Comput. Sci. 19 (5), 1–15. 10.1007/s11704-024-40072-y

[B20] Kubiniok P. Marcu A. Bichmann L. Kuchenbecker L. Schuster H. Hamelin D. J. (2022). Understanding the constitutive presentation of MHC class I immunopeptidomes in primary tissues. iScience 25 (2), 103768. 10.1016/j.isci.2022.103768 35141507 PMC8810409

[B21] Kulyyassov A. (2022). UNIPROT DATABASE - UNIVERSAL INFORMATION RESOURCE OF PROTEIN SEQUENCES. Eurasian J. Appl. Biotechnol. 10.11134/btp.1.2022.1

[B22] Li Y. Niu M. Zou Q. (2019). ELM-MHC: an improved MHC identification method with extreme learning machine algorithm. J. Proteome Research 18 (3), 1392–1401. 10.1021/acs.jproteome.9b00012 30698979

[B23] Li H. Pang Y. Liu B. (2021). BioSeq-BLM: a platform for analyzing DNA, RNA, and protein sequences based on biological language models. Nucleic Acids Res. 49 (22), e129. 10.1093/nar/gkab829 34581805 PMC8682797

[B24] Liu B. Gao X. Zhang H. (2019). BioSeq-Analysis2.0: an updated platform for analyzing DNA, RNA and protein sequences at sequence level and residue level based on machine learning approaches. Nucleic Acids Res. 47 (20), e127. 10.1093/nar/gkz740 31504851 PMC6847461

[B25] Luo Y. Shi L. Li Y. Zhuang A. Gong Y. Liu L. (2025). From intention to implementation: automating biomedical research *via* LLMs. Sci. China Inf. Sci. 68 (7), 170105. 10.1007/s11432-024-4485-0

[B26] Middleton D. (2005). HLA typing from serology to sequencing era. Iran. Journal Allergy, Asthma, Immunology 4, 53–66. 17301424

[B27] Mohapatra M. Sahu C. Mohapatra S. (2025). Trends of artificial intelligence (AI) use in drug targets, discovery and development: current status and future perspectives. Curr. Drug Targets 26 (4), 221–242. 10.2174/0113894501322734241008163304 39473198

[B28] Mu Q. Yu G. Zhou G. He Y. Zhang J. (2025). DRBP-EDP: classification of DNA-binding proteins and RNA-binding proteins using ESM-2 and dual-path neural network. NAR Genomics and Bioinforma. 7 (2). 10.1093/nargab/lqaf058 40391089 PMC12086546

[B30] Nallapareddy M. V. Dwivedula R. (2021). ABLE: attention based learning for enzyme classification. Comput. Biol. Chem. 94, 107558. 10.1016/j.compbiolchem.2021.107558 34481129

[B31] Neefjes J. Jongsma M. L. Paul P. Bakke O. (2011). Towards a systems understanding of MHC class I and MHC class II antigen presentation. Nat. Rev. Immunol. 11 (12), 823–836. 10.1038/nri3084 22076556

[B17] Pajila P. B. Sheena B. G. Gayathri A. Aswini J. Nalini M. (2023). “A comprehensive survey on naive bayes algorithm: advantages, limitations and applications,” in 2023 4th International Conference on Smart Electronics and Communication (ICOSEC). IEEE, 1228–1234.

[B32] Pawar P. (2025). A novel framework for protein sequence classification using LSTM and CNN. J. Inf. Syst. Eng. Manag. 10 (9s), 526–535. 10.52783/jisem.v10i9s.1251

[B34] Qiao J. Jin J. Yu H. Wei L. (2024). Towards retraining-free RNA modification prediction with incremental learning. Inf. Sci. 660, 120105. 10.1016/j.ins.2024.120105

[B35] Ren R. Ren R. (2025). Fhpg:A unified framework for transformer with pruning and quantization. 10.2139/ssrn.5123268

[B36] Rives A. Meier J. Sercu T. Goyal S. Lin Z. Liu J. (2021). Biological structure and function emerge from scaling unsupervised learning to 250 million protein sequences. Cold Spring Harb. Lab. 118 (15), e2016239118. 10.1073/pnas.2016239118 33876751 PMC8053943

[B37] Souza T. (2025). *Principal component analysis (PCA)*. 2025: Principal component analysis (PCA).

[B38] Soylu N. N. Sefer E. (2024). DeepPTM: protein post-translational modification prediction from protein sequences by combining deep protein language model with vision transformers. Curr. Bioinforma. 19 (9), 810–824. 10.2174/0115748936283134240109054157

[B39] Tien Bui D. Ho T. C. Pradhan B. Pham B. T. Nhu V. H. Revhaug I. (2016). GIS-based modeling of rainfall-induced landslides using data mining-based functional trees classifier with AdaBoost, Bagging, and MultiBoost ensemble frameworks. Environ. Earth Sci. 75 (14), 1–22. 10.1007/s12665-016-5919-4

[B40] Trowsdale J. Knight J. C. (2013). Major histocompatibility complex genomics and human disease. Annu. Rev. Genomics and Hum. Genet. 14 (1), 301–323. 10.1146/annurev-genom-091212-153455 23875801 PMC4426292

[B41] Tsai S. Santamaria P. (2013). MHC class II polymorphisms, autoreactive T-cells, and autoimmunity. Front. Immunol. 4, 321. 10.3389/fimmu.2013.00321 24133494 PMC3794362

[B42] Usman M. Lee J. A. (2019). “AFP-CKSAAP: prediction of antifreeze proteins using composition of k-Spaced amino acid pairs with deep neural network,” in 2019 IEEE 19th international conference on bioinformatics and bioengineering (BIBE), 38–43. 10.1109/bibe.2019.00016

[B43] Vaswani A. Shazeer N. Parmar N. Uszkoreit J. Jones L. Gomez A. N. (2017). Attention is all you need. Advances in Neural Information Processing Systems, 30.

[B44] Wang R. Jiang Y. Jin J. Yin C. Yu H. Wang F. (2023). DeepBIO: an automated and interpretable deep-learning platform for high-throughput biological sequence prediction, functional annotation and visualization analysis. Nucleic Acids Res. 51 (7), 3017–3029. 10.1093/nar/gkad055 36796796 PMC10123094

[B45] Wang Y. Zhai Y. Ding Y. Zou Q. (2024). SBSM-Pro: support bio-sequence machine for proteins. Sci. China Inf. Sci. 67 (11), 212106. 10.1007/s11432-024-4171-9

[B46] Wassenaar T. M. Harville T. Chastain J. Wanchai V. Ussery D. W. (2024). DNA structural features and variability of complete MHC locus sequences. Front. Bioinforma. 4, 1392613. 10.3389/fbinf.2024.1392613 39022183 PMC11251971

[B47] Watanabe S. Leow C. S. Hoshino J. Utsuro T. Nishizaki H. (2024). “Assessment and improvement of customer service speech with multiple large language models,” in Asia Pacific Signal and Information Processing Association Annual Summit and Conference (APSIPA ASC), 2024, 1–6. 10.1109/apsipaasc63619.2025.10849072

[B48] Wisky I. A. Defit S. Nurcahyo G. W. (2024). “A hybrid method of N-Gram and Bag-of-Words (BoW) models on the assessment of adolescent personality traits through the application of the naïve bayes algorithm,” in 2024 International Conference on Future Technologies for Smart Society (ICFTSS). IEEE, 57–62.

[B49] Wu S. Xu J. Guo J. T. (2025). Accurate prediction of nucleic acid binding proteins using protein language model. Bioinforma. Adv. 5 (1), vbaf008. 10.1093/bioadv/vbaf008 39990254 PMC11845279

[B50] Xie H. Wang L. Qian Y. Ding Y. Guo F. (2025). Methyl-GP: accurate generic DNA methylation prediction based on a language model and representation learning. Nucleic Acids Res. 53 (6), gkaf223. 10.1093/nar/gkaf223 40156859 PMC11952970

[B51] Xie X. Wu C. Dao F. (2025). scRiskCell: a single-cell framework for quantifying pancreatic islet risk cells and unravelling their dynamic transcriptional and molecular adaptation in the progression of type 2 diabetes. iMeta, e70060. 10.1002/imt2.70060 40860447 PMC12371254

[B52] Xu L. Science I. (2024). Deep learning for protein-protein contact prediction using evolutionary scale modeling (ESM) feature. Commun. Comput. Inf. Sci. 98–111. 10.1007/978-981-97-1277-9_8

[B53] Yadav S. Vora D. S. Sundar D. Dhanjal J. K. (2024). TCR-ESM: employing protein language embeddings to predict TCR-peptide-MHC binding. Comput. Struct. Biotechnol. J. 23, 9–173. 10.1016/j.csbj.2023.11.037 38146434 PMC10749252

[B54] Yan K. Lv H. Shao J. Chen S. Liu B. (2024). TPpred-SC: multi-functional therapeutic peptide prediction based on multi-label supervised contrastive learning. Sci. China Inf. Sci. 67 (11), 212105. 10.1007/s11432-024-4147-8

[B33] Yuan Q. Tian C. Song Y. Ou P. Zhu M. Zhao H. (2024). GPSFun: geometry-aware protein sequence function predictions with language models. Nucleic Acids Res. 52 (W1), W248–W255. 10.1093/nar/gkae381 38738636 PMC11223820

[B55] Zeng T. Wang Y. Tang B. Cui H. Tang D. Ding H. (2025). Colorectal liver metastasis pathomics model (CLMPM): integrating single cell and spatial transcriptome analysis with pathomics for predicting liver metastasis in colorectal cancer. Mod. Pathol. 38, 100805. 10.1016/j.modpat.2025.100805 40473111

[B56] Zhu W. Yuan S. S. Li J. Huang C. B. Lin H. Liao B. (2023). A first computational frame for recognizing heparin-binding protein. Diagn. (Basel) 13 (14), 2465. 10.3390/diagnostics13142465 37510209 PMC10377868

[B57] Zhu H. Hao H. Yu L. (2024). Identification of microbe–disease signed associations *via* multi-scale variational graph autoencoder based on signed message propagation. BMC Biology 22 (1), 172. 10.1186/s12915-024-01968-0 39148051 PMC11328394

[B58] Zou X. Ren L. Cai P. Zhang Y. Ding H. Deng K. (2023). Accurately identifying hemagglutinin using sequence information and machine learning methods. Front. Med. (Lausanne) 10, 1281880. 10.3389/fmed.2023.1281880 38020152 PMC10644030

[B59] Zou K. Wang Z. Zhu S. Wang S. Yang F. (2023). IDRnet: a novel pixel-enlightened neural network for predicting protein subcellular location based on interactive pointwise attention. Curr. Bioinform. 18 (10), 805–816.

[B60] Zulfiqar H. Guo Z. Ahmad R. M. Ahmed Z. Cai P. Chen X. (2024). Deep-STP: a deep learning-based approach to predict snake toxin proteins by using word embeddings. Front. Med. 10, 1291352. 10.3389/fmed.2023.1291352 38298505 PMC10829051

